# FAMILY CAREGIVER COMFORT WITH TELEHEALTH TECHNOLOGIES: DIFFERENCES BY RACE/ETHNICITY IN A CROSS SECTIONAL SURVEY

**DOI:** 10.1093/geroni/igad104.3348

**Published:** 2023-12-21

**Authors:** Reed Bratches, Wambui Onsando, Frank Puga, J Nicholas Odom, Paul Barr

**Affiliations:** University of Alabama at Birmingham, Birmingham, Alabama, United States; Dartmouth College, Hanover, New Hampshire, United States; University of Alabama at Birmingham, Birmingham, Alabama, United States; University of Alabama at Birmingham, Birmingham, Alabama, United States; Dartmouth College, Hanover, New Hampshire, United States

## Abstract

Telehealth has seen widespread use since the onset of the COVID-19 pandemic, and 82% patients required assistance in accessing their telehealth appointments. This assistance commonly comes from a family caregiver who may or may not be comfortable using the technologies associated with telehealth. The objective of our study was to analyze a demographically representative survey of US family caregivers to understand the level of comfort using telehealth technologies among family caregivers through a secondary analysis of survey data collected during the COVID-19 pandemic in 2020. Level of caregiver comfort using computers, smartphones, and tablets was determined through three Likert-style questions. Proportional odds logistic regression was used to understand the associations between demographic variables and level of caregiver comfort using each technology, when adjusting for covariates. A total of 340 caregivers were included in the analysis. Compared to Non-Hispanic White caregivers, Asian caregivers had a higher odds (OR 3.14; 95%CI 1.36, 8.02; p=0.01) of expressing comfort using computers; Black caregivers (OR 0.46; 95%CI 0.21, 0.98; p=0.04) and Hispanic caregivers (OR 0.36; 95%CI 0.17, 0.79 p=0.01) expressed lower odds of comfort using smartphones; and Asian caregivers had a higher odds (OR 4.64; 95%CI 2.05, 11.69; p=0.001) of expressing comfort using tablets. : There are identified disparities in level of technological comfort using computers, smartphones, and tablets by different racial and ethnic groups. Health systems should consider early stakeholder involvement in the design of telehealth technologies and culturally responsive training materials on telehealth technology use to reduce disparities in comfort using telehealth technologies.

